# Pre- and Post-natal High Fat Feeding Differentially Affects the Structure and Integrity of the Neurovascular Unit of 16-Month Old Male and Female Mice

**DOI:** 10.3389/fnins.2019.01045

**Published:** 2019-10-02

**Authors:** Laura Contu, Shereen Nizari, Christopher J. Heath, Cheryl A. Hawkes

**Affiliations:** School of Life, Health and Chemical Sciences, Faculty of Science, Technology, Engineering and Mathematics, The Open University, Milton Keynes, United Kingdom

**Keywords:** maternal obesity, aged, high fat diet, cerebrovascular, sex difference

## Abstract

Compelling experimental and clinical evidence supports a role for maternal obesity in offspring health. Adult children of obese mothers are at greater risk of obesity, diabetes, coronary heart disease and stroke. These offspring may also be at greater risk of age-related neurodegenerative diseases for which mid-life obesity is a risk factor. Rodent diet-induced obesity models have shown that high fat (HF) diet consumption damages the integrity of the blood–brain barrier (BBB) in the adult brain. However, there is currently little information about the effect of chronic HF feeding on the BBB of aged animals. Moreover, the long-term consequences of maternal obesity on the cerebrovasculature of aged offspring are not known. This study determined the impact of pre- and post-natal HF diet on the structure and integrity of cerebral blood vessels in aged male and female mice. Female C57Bl/6 mice were fed either a 10% fat control (C) or 45% HF diet before mating and during gestation and lactation. At weaning, male and female offspring were fed the C or HF diet until sacrifice at 16-months of age. Both dams and offspring fed the HF diet weighed significantly more than mice fed the C diet. Post-natal HF diet exposure increased hippocampal BBB leakiness in female offspring, in association with loss of astrocyte endfoot coverage of arteries. Markers of tight junctions, pericytes or smooth muscle cells were not altered by pre- or post-natal HF diet. Male offspring born to HF-fed mothers showed decreased parenchymal GFAP expression compared to offspring of mothers fed C diet, while microglial and macrophage markers were higher in the same female diet group. In addition, female offspring exposed to the HF diet for their entire lifespan showed more significant changes in vessel structure, BBB permeability and inflammation compared to male animals. These results suggest that the long-term impact of prenatal HF diet on the integrity of cerebral blood vessels differs between male and female offspring depending on the post-natal diet. This may have implications for the prevention and management of age- and obesity-related cerebrovascular diseases that differentially affect men and women.

## Introduction

Rates of obesity have risen consistently over the past three decades (Ng et al., 2014) in association with increasingly sedentary lifestyles and consumption of diets that are high in saturated fat (Corella et al., 2011; Phillips et al., 2012). Global obesity of women aged 20 years and older is currently estimated to be around 30%, with prevalence close to or over 60% in some countries (Ng et al., 2014). Rates of maternal obesity are also on the rise (Kim et al., 2007; Heslehurst et al., 2010; Gregor et al., 2016), due to both greater numbers of obese pregnant women and excess weight gain during pregnancy (Siega-Riz and Gray, 2013; Lindberg et al., 2016).

The developmental origins of health and disease (DoHAD) hypothesis posits that fetal adaptations in response to the early life environment have long-term consequences on health and alter the relative risk of developing diseases in later life. In particular, it is suggested that alterations made during the prenatal period to promote fetal wellbeing can become maladaptive if there is a mismatch between the pre- and post-natal environments (Wadhwa et al., 2009). There is now a significant body of clinical and experimental evidence supporting an influence of maternal obesity on the health of adult offspring, including higher body-mass index (Hochner et al., 2012; Eriksson et al., 2015) and increased risk of coronary heart disease, diabetes, stroke, asthma and premature death (Reynolds et al., 2013; Parlee and MacDougald, 2014; Godfrey et al., 2017). However, few studies have examined the impact of maternal obesity on offspring health beyond middle age.

Age is a major risk factor for the development of neurodegenerative diseases in which there is cerebrovascular dysfunction, including stroke, vascular dementia and Alzheimer’s disease (AD) (Sweeney et al., 2018). Modifiable conditions such as diabetes, hypertension and obesity also increase the risk of developing these diseases (Kivipelto et al., 2002; Reitz and Mayeux, 2014). The prevalence of stroke and AD is higher in aged women than men and the risk is increased after the onset of menopause (Persky et al., 2010; Lisabeth and Bushnell, 2012).

Cerebral blood vessels are composed of endothelial cells, basement membrane proteins, pericytes, smooth muscle cells, astrocytes and neurons that are collectively referred to as the neurovascular unit (NVU). The NVU is characterized by the expression of the blood–brain barrier (BBB) which is formed and maintained by tight junctions, pericytes and astrocytes and acts as a barrier to the unregulated entry of peripheral components into the brain (Daneman and Prat, 2015). Breakdown of the BBB is a major complication of cerebrovascular accidents and may contribute to the pathophysiology of AD (Daneman and Prat, 2015).

Reports from animal studies suggest that high fat diet consumption causes damage to the BBB, including increased leakiness, downregulation of tight junctions and cytoskeletal proteins and loss of pericyte coverage in brain areas such as the hypothalamus and hippocampus (Kanoski et al., 2010; Davidson et al., 2012; Pallebage-Gamarallage et al., 2012; Hargrave et al., 2016; Hajiluian et al., 2017; Mamo et al., 2019; Salameh et al., 2019). Loss of BBB integrity in late life has also been associated with mid-life obesity in humans (Gustafson et al., 2007). There is also evidence to suggest that BBB damage is exacerbated in aged mice fed a HF diet (Tucsek et al., 2014a, b). However, no studies have examined the effect of chronic (e.g., >1 year) high fat feeding on the cerebrovasculature of aged animals. The purpose of this study was to determine the long-term impact of pre- and post-natal high fat feeding on the structure and integrity of the NVU and BBB in 16-month old male and female mice.

## Materials and Methods

### Animal Model

Proven female C57Bl/6 breeders were fed either a control (C, 10% kcal fat, 20% kcal protein, 70% kcal carbohydrate, *n* = 11) or high fat (HF, 45% kcal fat, 20% kcal protein, 35% kcal carbohydrate; Special Diet Services, United Kingdom, *n* = 11) diet for 4 weeks before mating and during gestation and lactation. Diets were isocaloric and matched for amino acid, macro minerals and vitamin composition (Supplementary Table S1). Studs were maintained on the C diet. At weaning, male and female offspring were assigned either the C or HF diet, generating four experimental groups (*n* = 9/group/sex): C/C, C/HF, HF/C, HF/HF representing the pre- and post-weaning diet. All offspring were maintained on the diet until sacrifice at 16 months of age, but underwent food restriction (to approximately 90% free feeding weight) for 3 months at 6- and 12-months of age as part of a separate behavioral study (Supplementary Figure S1A). At sacrifice, weight-to-length ratio was calculated by dividing body weight (g) by nasal-anal distance (cm). Gonadal fat pad weight was also recorded for the offspring. All experiments were reviewed and approved by the Open University Animal Welfare and Ethics Review Board and the Home Office as per the UK Animal (Scientific Procedures) Act 1986 Amendment Regulations 2012 (PPL 70/8507).

### Western Blotting

Mice were deeply anesthetized with an overdose of sodium pentobarbital and perfused intracardially with 0.01 M phosphate buffered saline (PBS). Brains were removed immediately, dissected into individual regions and snap frozen. Hippocampal tissues from C/C, C/HF, HF/C and HF/HF mice (*n* = 4/group/sex) were homogenized in Ripa lysis buffer [20 mM Tris–HCl (pH 8.0), 150 mM NaCl, 1 mM EDTA, 0.1% SDS, 1% Igepal, 50 mM NaF, 1 mM NaVO3] containing a protease inhibitor cocktail (Merck Millipore, Watford, United Kingdom), spun down (13,000 g, 10 min, 4°C) and supernatants were frozen at –80°C until further use. Proteins (10–45 μg) were separated by gel electrophoresis on 4–20% Tris–glycine or 10% Tris–tricine gels (Fisher Scientific, Loughborough, United Kingdom) and transferred onto a nitrocellulose membrane. Membranes were incubated overnight at 4°C with primary antibodies against markers of the NVU (Table 1). Blots were stripped and re-probed with anti-glyceraldehyde-3-phosphate dehydrogenase (GAPDH, 1:50,000, Sigma-Aldrich) antibody to ensure equal protein loading. Two blots were replicated for each antibody. Immunoblots were quantified by densitometry using ImageJ (NIH, MD, United States) and calculated as an optical density ratio of protein levels normalized to GAPDH levels.

**TABLE 1:** Antibodies used in Western blotting (WB) or immunohistochemistry (IH) to label the NVU.

**NVU Protein**	**Antibody**	**Dilution**	**Source**
Endothelial cell	Anti-CD31	1:100	BD Biosciences, Wokingham, United Kingdom
Tight junction	Anti- zona occludens-1 (ZO-1) Anti-claudin 5	1:300 1:500	Abcam, Cambridge, United Kingdom Fisher Scientific, Loughborough, United Kingdom
Pericyte	Anti-platelet-derived growth factor receptor-β (PDGFRβ)	1:200	R&D Systems, Abingdon, United Kingdom
Smooth muscle cell	Anti-α smooth muscle actin (α-SMA) Anti-α-SMA-FITC	1:350 (WB) 1:350 (IH)	Sigma-Aldrich, Poole, United Kingdom Sigma-Aldrich, Poole, United Kingdom
Basement membrane	Anti-laminin	1:500 (WB) 1:350 (IH)	Sigma-Aldrich, Poole, United Kingdom
Astrocyte	Anti-glial fibrillary acidic protein (GFAP)	1:5000 (WB) 1:1000 (IH)	Agilent Technologies, Stockport, United Kingdom Abcam, Cambridge, United Kingdom

### Immunohistochemistry and Staining

Mice were perfused intracardially with 0.01 M PBS followed by 4% paraformaldehyde (*n* = 5/group/sex). Brains were sectioned on a cryostat (20 μm thickness), collected in a free-floating manner and stored at –20°C. For single-labeling immunohistochemistry, tissue sections were washed in 0.01 M PBS, blocked with 3% hydrogen peroxide and 15% normal goat serum (Sigma-Aldrich) and incubated with anti-ionized calcium binding adaptor molecule 1 (Iba1, 1:500, Alpha Labs, Eastleigh, United Kingdom), potato lectin (*Solanum tuberosum*, 1:500, Vector Labs, Peterborough, United Kingdom) or biotinylated anti-mouse (1:500, Vector Labs). Sections were incubated with anti-rabbit (1:400, Vector Labs) and/or avidin-biotin complex (1:200, Vector Labs) and developed using glucose oxidase enhancement with DAB as the chromogen.

For Iba1 and CD68 co-localization, sections were treated with boiling sodium citrate buffer (10 mM containing 0.1% Triton X-100) and incubated overnight with anti-Iba1 (1:350) and anti-CD68 (1:500, Biorad, Watford, United Kingdom). Sections were then developed with anti-rabbit AlexaFluor 555 (1:200, Fisher Scientific) and anti-rat AlexaFluor 633 (1:200, Fisher Scientific). For NVU labeling, sections were treated with pepsin (1 mg/mL in 0.2N HCl, 30 s at 37°C) and incubated overnight with anti-laminin and anti-GFAP (Table 1). The next day the sections were washed in PBS and incubated with FITC-conjugated anti-α-SMA (1:350, Sigma-Aldrich), anti-mouse AlexaFluor 405 (1:200, Fisher Scientific), anti-rabbit AlexaFluor 555 (1:200, Fisher Scientific) and anti-chicken AlexaFluor 633 (1:200, Fisher Scientific). Sections were coverslipped using Mowiol^®^ mounting media (Sigma-Aldrich) containing 0.1% v/v Citifluor (Citifluor Ltd., London, United Kingdom).

### Image Acquisition and Analysis

Non-overlapping DAB images were captured across the entire hippocampal formation or median eminence using a ×10 objective on a Nikon Eclipse 80 Brightfield Microscope. Non-overlapping images of fluorescent immunohistochemistry were taken using a Leica SP5 scanning laser confocal microscope. Brightfield images and individual channels from confocal images were quantified by calculating the percentage area covered by staining, total cell count and cell size using Fiji (NIH, MD, United States). For quantification of CD68 + microglia, the rolling ball radius was set at 2–15 μm^2^, while quantification of CD68 + macrophages were detected using a rolling ball radius of 16 μm^2^-infinity. For 3D reconstruction, confocal images were deconvolved using AutoQuant X3 (MediaCybernetics Inc., Rockville, MD). Deconvolved images were processed using Imaris (Bitplane^®^) and surfaces were created for laminin and GFAP. To quantify the amount of astrocyte endfoot contact with laminin at hippocampal arteries (defined as positive for α-SMA and >10 μm diameter), the total area of contact between GFAP and laminin (μm^2^) was calculated using the Imaris Xtension “Surface to Surface Contact Area” (Imaris V8.31, ImarisXT Bitplane Inc., created by Matthew J. Gastinger, Bitplane) and standardized to vessel diameter and length (μm). Only surfaces that made direct contact with each other (i.e., 0 μm distance) were quantified. GFAP-to-laminin contact was calculated for three randomly selected arteries for each mouse (*n* = 5/group/sex) and the average values per mouse were used for statistical analysis.

### Statistical Analysis

Data were confirmed to be normally distributed using the Kolmogorov–Smirnov test. The ROUT test was used to identify and exclude outliers. All analyses were carried out using two-way ANOVA with Holm–Sidak multiple comparisons *post hoc* test (GraphPad Prism, San Diego, CA, United States). Data represent mean ± SEM and *p* < 0.05 was considered to be statistically significant. Significant differences between C/C vs. HF/HF and C/HF vs. HF/C groups were considered biologically irrelevant and were not reported.

## Results

### High Fat Diet Results in Weight Gain in Mothers and Aged Offspring

**FIGURE 1:**
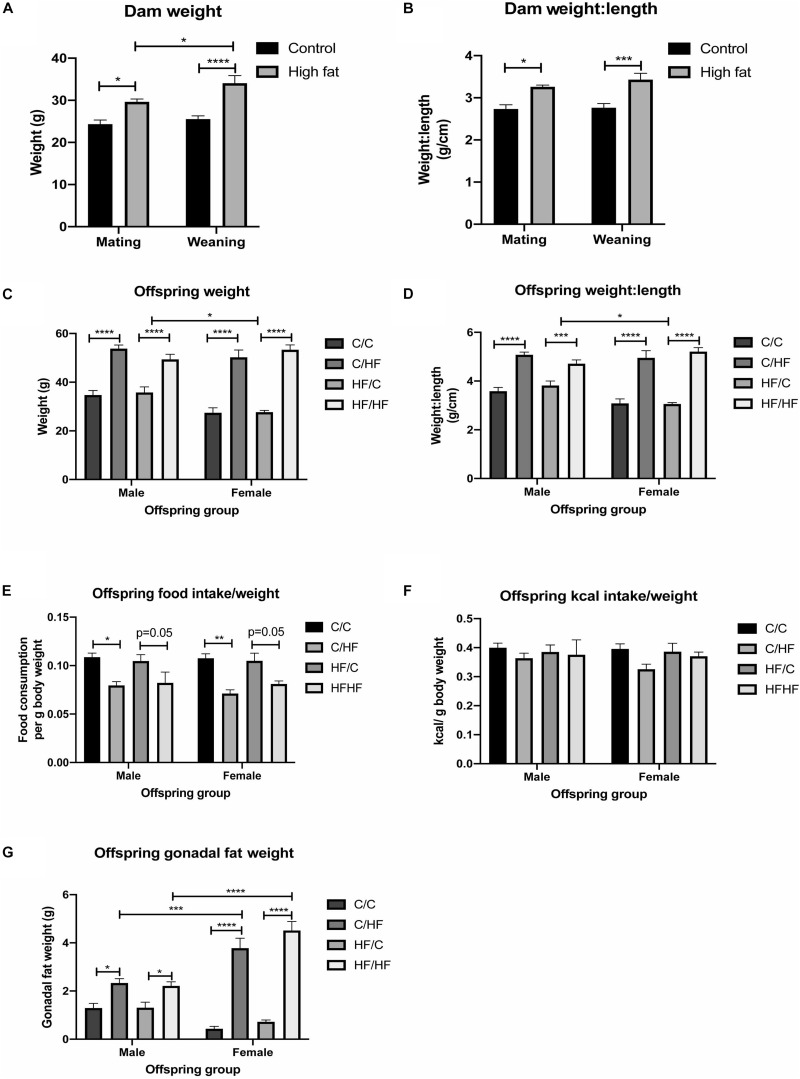
Effect of pre- and post-natal high fat feeding on food intake, body weight and gonadal fat. **(A,B)** Body weight **(A)** and weight-to-length ratio **(B)** of dams fed the control (C) or high fat (HF) diet for 4 weeks before mating and at weaning. **(C,D)** Body weight **(C)** and weight-to-length ratio **(D)** of 16-month old male and female C/C, C/HF, HF/C and HF/HF offspring. **(E,F)** Average daily food **(E)** and kcal **(F)** intake per gram body weight of 16-month old offspring diet groups. **(G)** Weight of gonadal fat for male and female C/C, C/HF, HF/C and HF/HF mice. Data represent mean ± SEM. ^∗^*p* < 0.05, ^∗∗^*p* < 0.01, ^∗∗∗^*p* < 0.001, ^****^p < 0.0001, two-way ANOVA with Sidak–Holm *post hoc* test.

Consumption of the HF diet resulted in a significantly greater weight and weight-to-length ratio of dams at both mating and weaning compared to dams fed the C diet (Figures 1A,B). 16-month old male and female C/HF and HF/HF offspring weighed significantly more than C/C and HF/C mice, respectively (Figure 1C). Weight-to-length ratio was also significantly greater in offspring fed the HF diet compared to those fed the C diet postnatally (Figure 1D). Changes in body weight were equivalent between diet groups during periods of food restriction and *ad libitum* consumption (Supplementary Figures S1B,C), although female HF/C mice gained significantly more weight after restriction than their male counterparts (Supplementary Figure S1C). Male and female HF-fed offspring weighed significantly more than C-fed mice throughout the 16-month period (Supplementary Figures S1D,E). 16-month old male HF/C offspring weighed significantly more than their female counterparts and there was a non-significant trend (*p* = 0.05) toward lower body weight in C/C female compared to C/C males (Figure 1C). However, both groups of female offspring fed the HF diet (i.e., C/HF and HF/HF) weighed the same and had the same weight-to-length ratio as males in the comparable diet groups (Figures 1C,D). Proportional to their body weight, HF-fed mice consumed less food than C-fed mice (Figure 1E), but kcal consumption was similar between diet groups (Figure 1F). Gonadal fat weight, which is related to total body fat (Rogers and Webb, 1980), was significantly higher in male and female C/HF and HF/HF groups compared to C/C and HF/C mice, respectively (Figure 1G). No differences in weight, weight-to-length ratio or gonadal fat were observed between offspring that were maintained on the same postnatal diet (i.e., C/C vs. HF/C or C/HF vs. HF/HF). Female C/HF and HF/HF mice also showed greater fat content than corresponding male mice (Figure 1G).

### Pre- and Post-natal Diet Does Not Affect the Level of NVU Components in Aged Offspring

**FIGURE 2:**
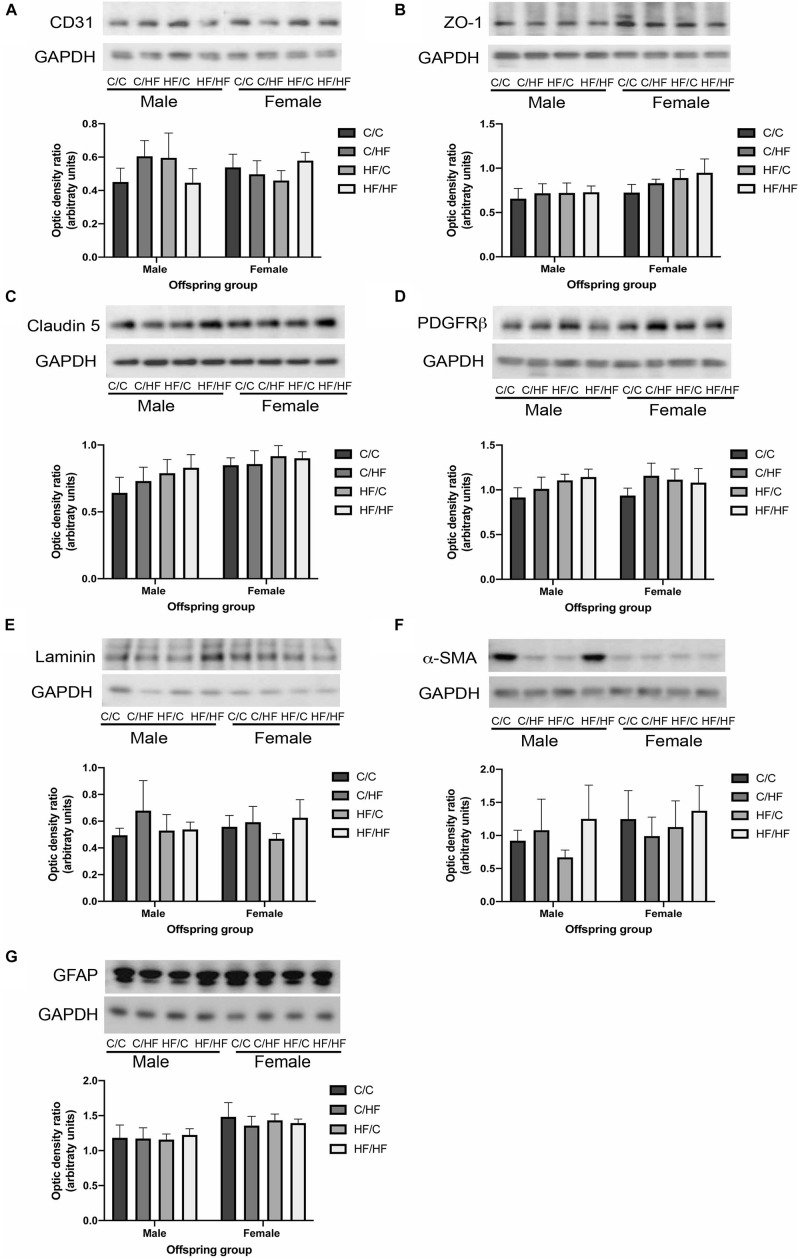
Expression of neurovascular unit components in aged offspring. **(A–G)** Hippocampal homogenates from 16-month old male and female C/C, C/HF, HF/C and HF/HF mice were assessed by Western blot to determine the expression of CD31 **(A)**, zona occludens-1 (ZO-1, **B**), claudin 5 **(C)**, platelet-derived growth factor receptor-β (PDGFRβ, **D**), laminin **(E)**, α-smooth muscle actin (α-SMA, **F**) and glial fibrillary acidic protein (GFAP, **G**). Data represent mean ± SEM.

To evaluate the long-term effect of pre- and post-natal high fat feeding on NVU components, hippocampal tissues from offspring were processed by Western blotting for markers of endothelial cells (CD31), tight junction proteins (ZO-1 and claudin 5), pericytes (PDGFRβ), cerebrovascular basement membranes (laminin), smooth muscle cells (α-SMA) and astrocytes (GFAP). As shown in Figure 2A, no differences were noted in the levels of CD31 between any diet group or between males and females. A trend toward increased expression of ZO-1 and claudin 5 was observed in female vs. male mice, but this did not reach statistical significance (*p* = 0.06 and *p* = 0.05, respectively) and no differences were noted between diet groups for either protein (Figures 2B,C). No effects of diet or sex were observed in the expression of PDGFRβ (Figure 2D), laminin (Figure 2E) or α-SMA (Figure 2F). Two-way ANOVA revealed a significant effect of sex on GFAP expression (*p* = 0.02), however, *post hoc* comparisons between individual male and female offspring groups did not reveal any significant differences (Figure 2G).

### Pre- and Post-natal High Fat Feeding Differentially Affects the Leakiness of the Hippocampal BBB Between Male and Female Offspring

**FIGURE 3:**
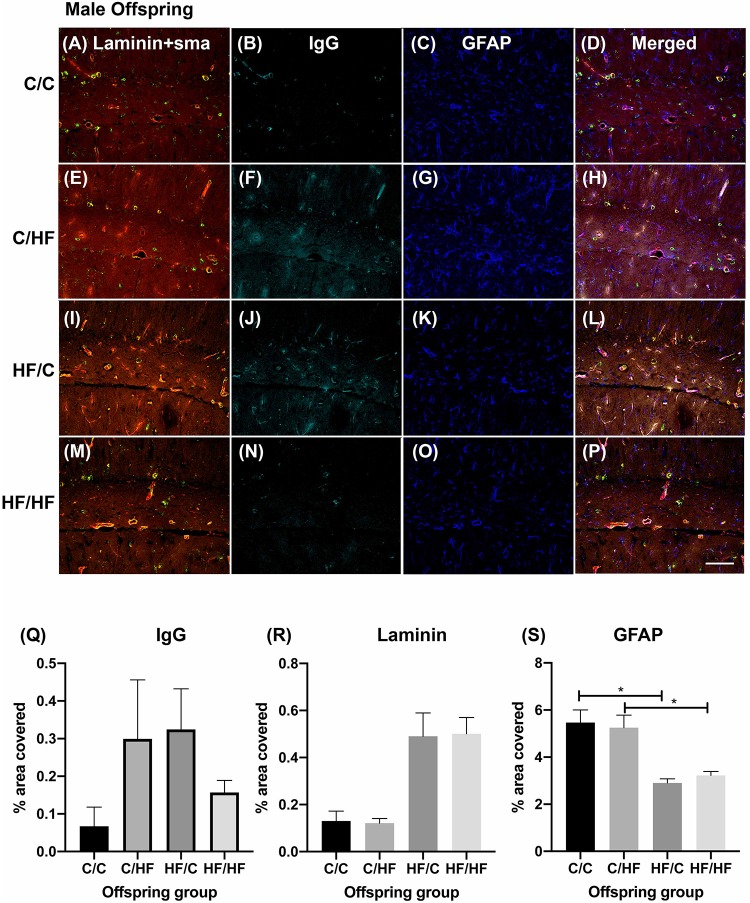
Effect of pre- and post-natal high fat diet on hippocampal blood–brain barrier integrity in male offspring. **(A–P)** Photomicrographs of hippocampal tissue sections stained for laminin (red, **A,E,I,M**), smooth muscle actin (green, **A,E,I,M**), IgG (turquoise, **B,F,J,N**) and GFAP (blue, **C,G,K,O**) in 16-month old male C/C **(A–D)**, C/HF **(E–H)**, HF/C **(I–L)** and HF/HF mice **(M–P)**. **(Q–S)** Quantification of hippocampal area positive for IgG extravasation **(Q)**, laminin **(R)** and GFAP **(S)**. Data represent mean ± SEM. ^∗^*p* < 0.05, two-way ANOVA with Holm–Sidak *post hoc* test. Scale bar = 100 μm.

To determine if BBB integrity was differentially affected between the offspring groups, tissue sections were processed using antibodies against laminin, α-SMA, GFAP and mouse IgG as markers of blood vessels, arteries, astrocytes and extravasated plasma proteins, respectively. Within the male offspring groups, BBB integrity appeared relatively intact in the C/C group, with little IgG expression detected in the parenchyma (Figures 3A–D). Diffuse IgG staining was detected around capillaries in the hippocampi of both C/HF and HF/C mice (Figures 3E–L), although the degree of IgG detection was variable between animals within both groups. Interestingly, HF/HF mice appeared to have less IgG staining than the C/HF and HF/C mice (Figures 3M–P). However, no statistically significant differences in the percent of hippocampal area positive for IgG were observed between diet groups (Figure 3Q). A similar pattern of detection was observed in tissues processed with biotinylated anti-mouse alone (Supplementary Figure S2A), confirming that the pattern of staining was not due to non-specific binding of IgG to laminin, α-SMA or GFAP. Analysis of laminin expression indicated increased expression in offspring of mothers fed the HF diet, however, this did not differ significantly from the other diet groups when analyzed by two-way ANOVA (Figure 3R). By contrast, GFAP expression as measured by cell count and area coverage was significantly lower in the hippocampus of HF/C and HF/HF mice compared to C/C and C/HF mice, respectively (Figure 3S and Supplementary Figure S3A). Average size of GFAP-positive astrocytes did not differ between diet groups (Supplementary Figure S3B).

**FIGURE 4:**
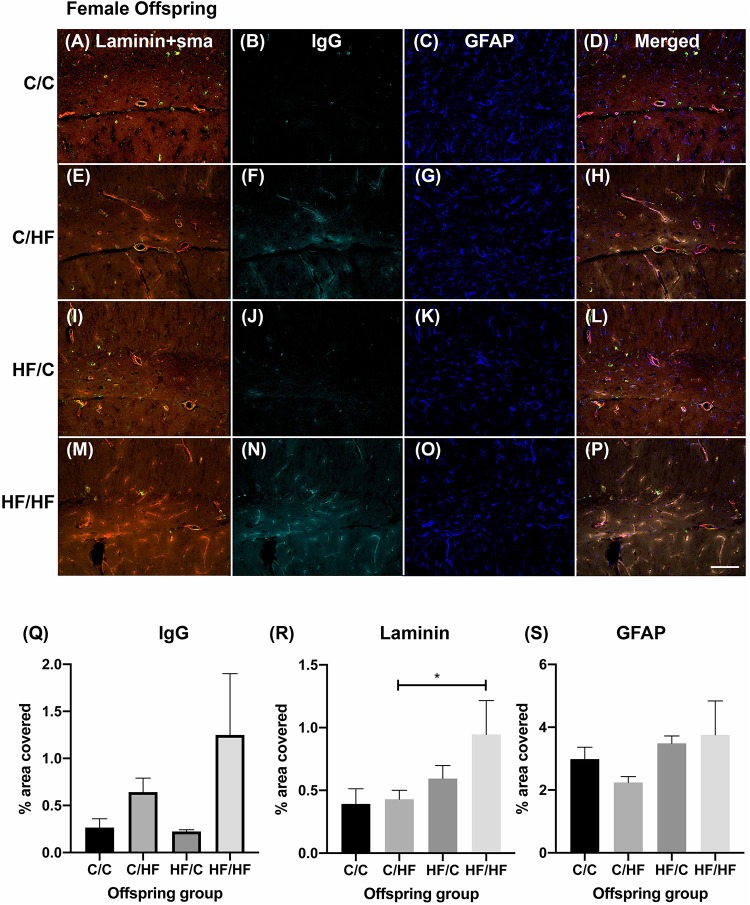
Effect of pre- and post-natal high fat diet on hippocampal blood–brain barrier integrity in female offspring. **(A)** Photomicrographs of hippocampal tissue sections stained for laminin (red, **A,E,I,M**), smooth muscle actin (green, **A,E,I,M**), IgG (turquoise, **B,F,J,N**) and GFAP (blue, **C,G,K,O**) in 16-month old male C/C **(A–D)**, C/HF **(E–H)**, HF/C **(I–L)** and HF/HF mice **(M–P)**. **(Q–S)** Quantification of hippocampal area positive for IgG extravasation **(Q)**, laminin **(R)** and GFAP **(S)**. Data represent mean ± SEM. ^∗^*p* < 0.05, two-way ANOVA with Holm–Sidak *post hoc* test. Scale bar = 100 μm.

Within the female offspring groups, IgG detection was minimal in the C/C group (Figures 4A–D) and appeared to be more prominent in the C/HF group (Figures 4E–H), although there was no statistically significant difference between the groups. The pattern of staining in HF/C mice was similar to that of C/C mice (Figures 4I–L). A large amount of IgG staining was observed in the hippocampi of HF/HF mice although intra-group variability was high (Figures 4M–P). Compared to HF/C mice, IgG coverage in HF/HF mice bordered statistical significance in quadruple-stained sections (*p* = 0.06) and was significantly higher in DAB-processed sections (Supplementary Figure S2A). IgG values did not differ significantly between HF/HF mice and the other diet groups (Figure 4Q). Laminin expression was significantly higher in the hippocampus of HF/HF mice compared to C/HF animals (Figure 4R). No significant differences in the coverage (Figure 4S), number (Supplementary Figure S3A) or size (Supplementary Figure S3B) of GFAP-positive astrocytes were noted between female offspring diet groups.

**FIGURE 5:**
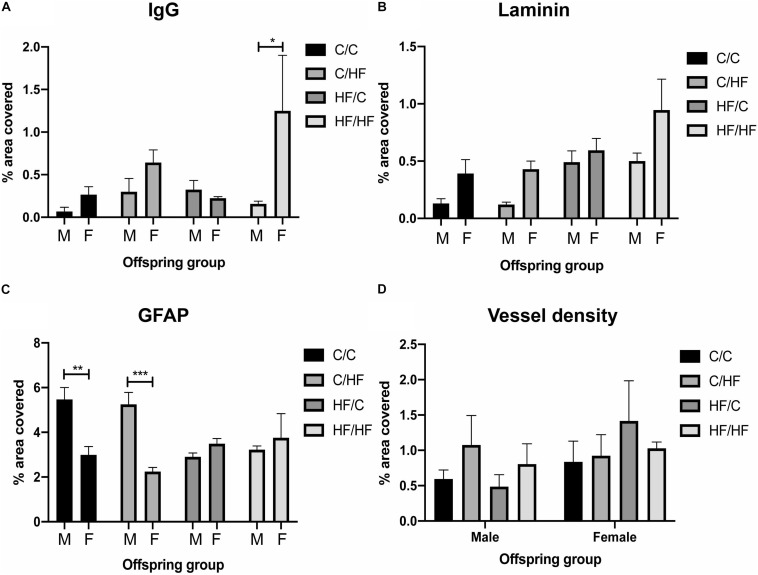
Comparison of blood–brain barrier integrity and vessel density between male and female offspring. **(A–C)** Quantification of hippocampal area positive for IgG extravasation **(A)**, laminin **(B)** and GFAP **(C)** in 16-month old male and female C/C, C/HF, HF/C and HF/HF mice. **(D)** Vessel density in hippocampus of male and female offspring. Data represent mean ± SEM. ^∗^*p* < 0.05, ^∗∗^*p* < 0.01, ^∗∗∗^*p* < 0.001, two-way ANOVA with Holm–Sidak *post hoc* test.

Comparisons between male and female offspring showed a trend toward increased IgG staining in female mice, which was significantly higher in HF/HF females compared to HF/HF males (Figure 5A and Supplementary Figure S2B). The expression of laminin followed a similar pattern but no statistically significant differences were noted between the sexes (Figure 5B). Both hippocampal coverage and cell counts of GFAP-positive astrocytes were significantly higher in male C/C and C/HF groups relative to the comparable female groups, while no differences were noted between males and females born to mothers fed the HF diet (Figure 5C and Supplementary Figure S3C). Average size of GFAP-positive astrocytes did not differ between male and female mice in any diet group (Supplementary Figure S3D). The density of potato lectin staining was similar between all diet groups and between male and female offspring (Figure 5D), suggesting that the observed differences in BBB leakiness and NVU expression were not due to differences in blood vessel density.

### Leakiness of the Median Eminence Is Not Affected by Pre- and Post-natal High Fat Feeding

**FIGURE 6:**
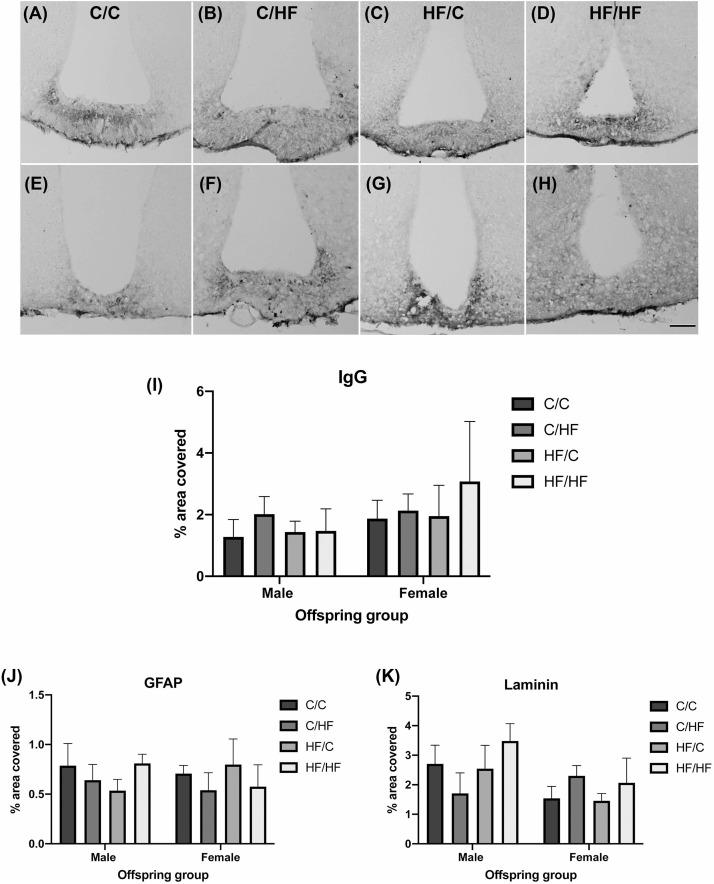
Effect of pre- and post-natal high fat diet on hypothalamic blood–brain barrier integrity in male and female offspring. **(A–H)** Photomicrographs of the median eminence stained for IgG in 16-month old male **(A–D)** and female **(E–H)** C/C **(A,E)**, C/HF **(B,F)**, HF/C **(C,G)** and HF/HF **(D,H)** mice. **(I)** Quantification of IgG extravasation in the median eminence. **(J,K)** Quantification of median eminence area positive for GFAP **(J)** and laminin **(K)**. Data represent mean ± SEM. Scale bar = 200 μm.

To determine if HF feeding also affected the NVU in an area with endogenous BBB leakiness, expression of IgG, GFAP and laminin was determined in the median eminence (ME). IgG staining was observed in the ME in male and female mice in all diet groups (Figures 6A–H). IgG coverage was similar between C and HF-fed mice and between males and females (Figure 6I). Hypothalamic areas surrounding the ME and arcuate nucleus did not show the presence of IgG, suggesting that stable IgG expression was not due to diffusion of IgG from the ME. Similarly, expression of GFAP (Figure 6J) and laminin (Figure 6K) did not differ significantly between diet groups.

### Prenatal High Fat Feeding Increases Inflammatory Markers in Aged Female Offspring

**FIGURE 7:**
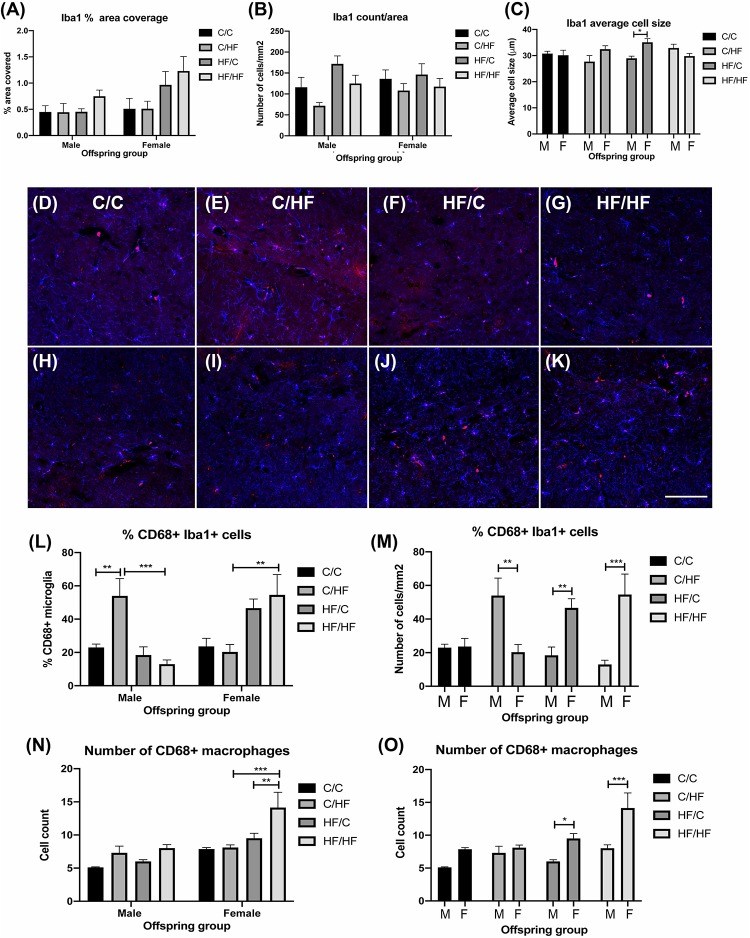
Evaluation of microglia and macrophage expression in the hippocampus of aged male and female offspring. **(A–C)** Quantification of the percent of hippocampal coverage **(A)**, cell count **(B)** and average size **(C)** of Iba1-positive microglia in 16-month old C/C, C/HF, HF/C and HF/HF mice. **(D–K)** Photomicrographs of hippocampal tissue sections stained for Iba1 (blue) and CD68 (red) in 16-month old male **(D–G)** and female **(H–K)** C/C **(D,H)**, C/HF **(E,I)**, HF/C **(F,J)** and HF/HF **(G,K)** mice. **(L,M)** Quantification of the percentage of Iba1-positive microglia that are also positive for CD68 within each diet group **(L)** and between male and female offspring **(M)**. **(N,O)** Quantification of the number of CD68-positive macrophages within each diet group **(N)** and between male and female offspring **(O)**. Data represent mean ± SEM. ^∗^*p* < 0.05, ^∗∗^*p* < 0.01, ^∗∗∗^*p* < 0.001, two-way ANOVA with Holm–Sidak *post hoc* test. Scale bar = 100 μm.

To evaluate if BBB leakiness and/or HF diet was associated with increased inflammation, sections were stained for the microglial marker Iba1. Two-way ANOVA showed a significant effect of diet (*p* = 0.03) and sex (*p* = 0.04) on% area coverage by Iba1, but *post hoc* tests were not significant between any groups, although comparison of female C/HF vs. HF/HF bordered significance (*p* = 0.05) (Figure 7A). Microglia number and average size did not differ between diet groups, however, microglia size was significantly higher in female HF/C mice compared to HF/C males (Figures 7B,C). To determine if microglia activity was altered, sections were double labeled with Iba1 and CD68 (Figures 7D–K), a macrophage marker that is also expressed by phagocytic microglia (Zotova et al., 2013). Quantification of the percentage of Iba1-positive microglia that were also positive for CD68 showed that C/HF male offspring expressed significantly more phagocytic microglia than male C/C and HF/HF mice (Figure 7L). The percent of CD68-positive microglia was also significantly higher in female HF/HF vs. C/HF mice (Figure 7L). Comparisons between male and female offspring showed that male C/HF mice had significantly higher expression of phagocytic microglia than C/HF females, however, CD68 expression in microglia was significantly higher in HF/C and HF/HF female mice compared to the comparable male diet groups (Figure 7M). The number of CD68-positive macrophages, which were located primarily in the perivascular spaces, was also significantly higher in HF/HF females compared to both female C/HF and HF/C mice (Figure 7N). Significantly more macrophages were also observed in female HF/C and HF/HF females compared to males in the same diet groups (Figure 7O).

### Perivascular Coverage by Astrocytes Is Decreased by Post-natal High Fat Feeding in Female but Not Male Offspring

**FIGURE 8:**
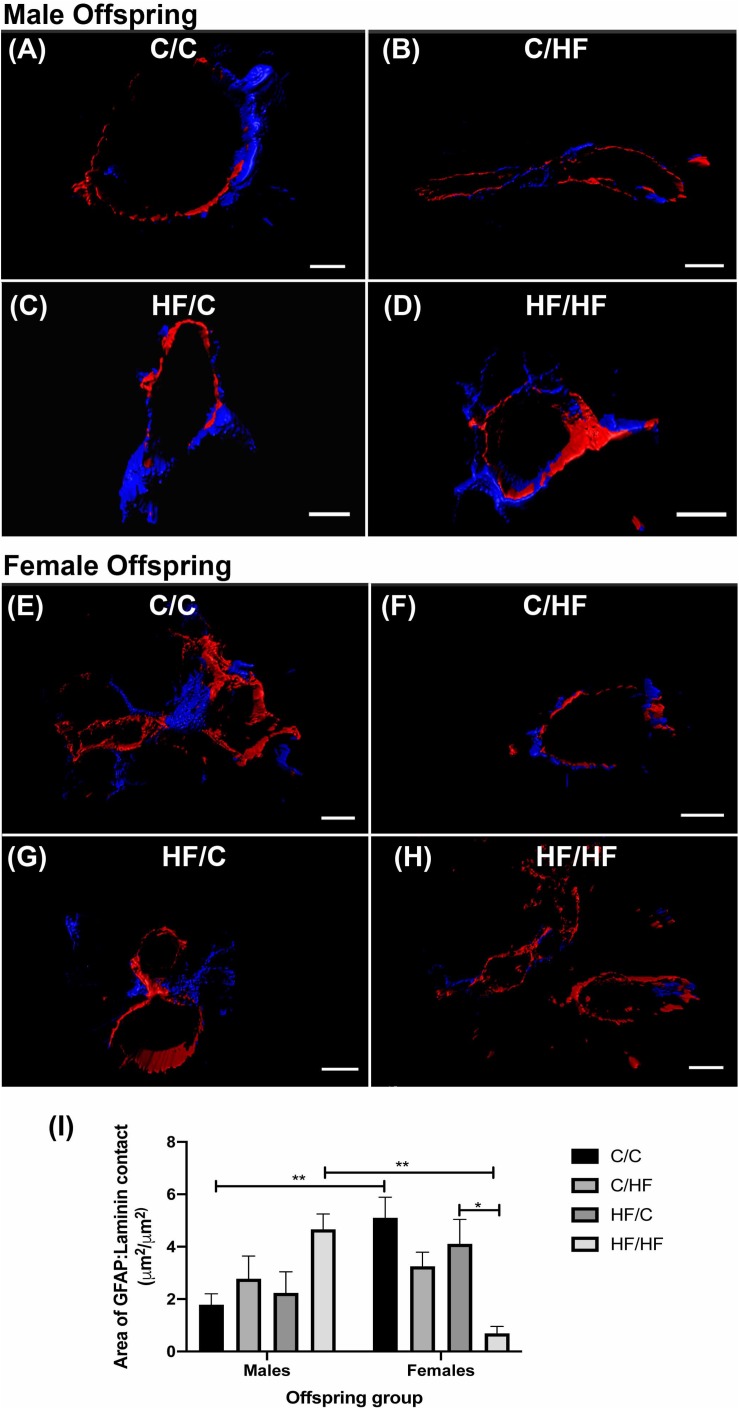
Astrocyte endfoot coverage of hippocampal arteries in male and female offspring. **(A–H)** Representative images of the 3D reconstruction of GFAP (blue) and laminin (red) staining of hippocampal arteries in male **(A–D)** and female **(E–H)** C/C **(A,E)**, C/HF **(B,F)**, HF/C **(C,G)** and HF/HF **(D,H)** mice. **(I)** Quantification of the surface area of contact between GFAP-positive astrocytes and laminin in arteries of male and female offspring. Data represent mean ± SEM. ^∗^*p* < 0.05, ^∗∗^*p* < 0.01, two-way ANOVA with Holm–Sidak *post hoc* test. Scale bars: **(A)** = 7 μm; **(B,D–F,H)** = 10 μm; **(C)** = 5 μm; **(G)** = 8 μm.

As astrocytes contribute to the maintenance of the BBB (Daneman and Prat, 2015), we assessed whether the observed differences in parenchymal GFAP expression were also observed at the NVU. The amount of direct surface area contact between astrocyte endfeet and laminin was analyzed using 3D reconstructions of hippocampal arteries from male (Figures 8A–D) and female (Figures 8E–H) offspring. Male offspring showed a pattern of increased astrocyte coverage in post-natal HF groups, with the highest amount of perivascular contact in the HF/HF group, although these differences did not differ significantly from the C groups (Figure 8I). Interestingly, the opposite pattern was detected in the female offspring, where astrocyte-to-laminin contact was significantly lower in HF/HF mice compared to HF/C animals (Figure 8I). Comparison between male and female offspring revealed that astrocyte endfoot coverage was significantly higher in C/C females compared to C/C males and significantly lower in HF/HF females versus the HF/HF male group.

## Discussion

Previous studies using HF feeding paradigms have typically assessed BBB structure in young adult animals after relatively short periods of feeding and none have to our knowledge evaluated the long-term effect of maternal obesity on the cerebrovasculature of aged offspring. The purpose of this study was to examine the impact of diet-induced maternal obesity on the structure and integrity of the NVU and BBB in aged offspring in the presence or absence of chronic HF feeding. We found that post-natal exposure to a HF diet resulted in increased leakiness of the BBB in the hippocampus of female offspring in association with loss of astrocyte endfoot coverage of hippocampal vessels. Male, but not female offspring born to mothers fed a HF diet also showed a trend toward increased BBB permeability and significantly decreased parenchymal GFAP expression compared to mice born to lean mothers. By contrast, female offspring exposed to the HF diet for their entire lifespan (i.e., HF/HF) exhibited more significant changes in NVU structure, BBB permeability and inflammation compared to their male counterparts.

Previous studies examining the effects of HF diet on the hippocampal BBB have reported increased permeability to circulating compounds (e.g., fluorescein, Evans blue, albumin, IgG) in rodents fed the diet over a 2–4 months period (Kanoski et al., 2010; Davidson et al., 2012; Pallebage-Gamarallage et al., 2012; Hargrave et al., 2016; Hajiluian et al., 2017; Mamo et al., 2019; Salameh et al., 2019). These findings are in agreement with our observations that chronic post-natal high fat feeding alone (i.e., C/HF) resulted in a trend toward increased extravasation of IgG into the hippocampus in both male and female offspring relative to C/C mice and significantly higher IgG content in HF/HF vs. HF/C females. We chose to use IgG extravasation as a proxy of BBB integrity because it is endogenously expressed and has a relatively large molecular weight (Saunders et al., 2015), suggesting that its presence in the parenchyma represents substantial damage to the BBB. However, this approach may have underestimated smaller changes in the tightness of the BBB and contributed to the intra-group variation that was observed.

Evaluation of the staining pattern of IgG in the median eminence, an area of endogenous BBB leakiness, revealed no effect of pre- or post-natal HF exposure on IgG content in the aged offspring. These findings are in contrast to previous reports of increased permeability of the hypothalamic BBB to albumin and glucose after 16 and 36 weeks of HF feeding (Salameh et al., 2019). Yi et al. (2012) reported increased IgG in the arcuate nucleus of mice fed a HF diet for 16 weeks, which was due to selective permeability to IgG1. The reasons for the discrepancy between the current findings and previous reports are unclear. It may be that chronic exposure to a HF diet from weaning induces compensatory changes in the hypothalamic BBB that counteract diet-induced damage or that the hypothalamic BBB of C/HF and HF/HF mice may be leakier to small molecular weight compounds (e.g., albumin) that were not evaluated in the current study. Alternatively, quantification of IgG staining using a general anti-mouse IgG that recognizes both heavy and light chains may have masked differences in selective permeability to specific IgG isotypes. Therefore, whether the observed changes in BBB permeability in the current study are specific to the hippocampus or other brain areas with an endogenously tight BBB requires further investigation.

While some BBB studies have reported decreased tight junction protein expression (Kanoski et al., 2010; de Aquino et al., 2018; Mamo et al., 2019) and reduced capillary density in the CA1 region of the hippocampus of HF-fed animals (Tucsek et al., 2014b), we did not observe differences in total levels of ZO-1 or claudin-5 or in vessel density between C/C and C/HF mice. As tight junction proteins are also expressed by neurons, astrocytes and oligodendrocytes (Bauer et al., 1999; Romanitan et al., 2010; Freeman and Granholm, 2012), it may be that subtle changes in the distribution or expression of tight junction proteins were masked in the analysis of whole hippocampal homogenates. Similarly, by analyzing vessel density across the entire hippocampus, region-specific changes between diet groups may have been overlooked. However, a previous study comparing BBB permeability in 24-month old mice fed a C or HF diet for 5 months reported increased levels of IgG in the hippocampus in HF-fed mice in the absence of differences in the expression of occludin or claudin-5 (Tucsek et al., 2014a). This suggests that alternative mechanism(s) may contribute to BBB leakiness in HF-fed aged mice. In support of this, we found that astrocyte endfoot contact with laminin in hippocampal arteries mirrored the pattern of IgG staining across the diet groups, in that areas of low astrocyte coverage were associated with higher parenchymal IgG and vice versa. Given that astrocytes contribute to the maintenance of the BBB (Daneman and Prat, 2015), this suggests that HF feeding may also contribute to BBB damage by directly influencing the degree of perivascular contact of astrocytes at the NVU, although whether this precedes or results from BBB dysfunction remains to be determined.

To date, few studies have examined the impact of prenatal high fat feeding or maternal obesity on the structure and function of the offspring NVU. A study by Kim et al. (2016a) found that fenestrations, endothelial transporters and tight junctions were significantly altered in the hypothalamic blood vessels of fetal and neonatal mice born to mothers fed a HF diet. We did not observe differences in IgG staining in the median eminence between 16-month old C/C and HF/C mice. As discussed above, whether this is due to post-natal adaptations during brain maturation or insensitivity of the current methods to detect small or selective changes in BBB permeability remains unknown. Endothelial denudation and thickening of the middle cerebral artery has been reported in 6-month old rat offspring of obese mothers (Lin et al., 2018) and neonatal and adult HF/C offspring display larger infarct volumes and poorer functional deficits after stroke compared to C/C rats (Lin et al., 2016; Teo et al., 2017). We have previously reported that vessel morphology and thickness and GFAP expression were altered in the hippocampus of 5-month old HF/C male mice compared to C/C offspring (Hawkes et al., 2015). In the present study, only parenchymal GFAP expression differed significantly between male C/C and HF/C mice, although there was also a trend toward increased expression of laminin and IgG extravasation in male HF/C offspring. Although previous studies have reported increased GFAP expression in the hypothalamus of fetal and neonatal mice born to obese mothers (Kim et al., 2016b), our findings are more consistent with the observation that astrocyte expression in male offspring born to mothers fed a hypercaloric drink was lower than those born to un-supplemented mothers (Molina et al., 2018). These findings, in combination with the stable expression of Iba1 as well as CD68 + microglia and macrophages between C/C and HF/C males, suggest that prenatal HF exposure does not induce a general gliosis in aged male offspring. Whether this results from an early adaptive mechanism against a pro-inflammatory developmental milieu (Molina et al., 2018) or is an aging-related phenomenon remains to be determined.

Differences between male and female mice were observed in almost every parameter of this study. Female offspring had a greater fat mass, a higher degree of IgG extravasation, lower expression of GFAP and higher expression of Iba1 and CD68 than male counterparts. The greater accumulation of visceral fat and increased BBB permeability in female mice are consistent with the effects of estrogen loss during reproductive senescence (Wilson et al., 2008; Bake et al., 2009; Shi and Clegg, 2009), which begins around 11 months of age in the C57Bl/6 strain (Nelson et al., 1981, 1982). In women and female rodents, estrogen appears to have an age-dependent effect on the health of the NVU and BBB function. In young rats, estrogen replacement improves BBB leakiness after ovariectomization but further exacerbates dye extravasation in reproductively senescent females (Bake and Sohrabji, 2004). Results from the Women’s Health Initiative study found that hormone replacement therapy in postmenopausal women increased the risk of stroke and the incidence of mild cognitive impairment and all cause dementia (Shumaker et al., 2004; Henderson and Lobo, 2012). Although levels of testosterone, the main source of estradiol in men, also decrease with age, estrogen levels remain constant in the aging male (Vermeulen et al., 2002). Thus, although increased levels of estradiol have been reported in both obese men and women (Schneider et al., 1979; Vermeulen et al., 2002), it is possible that the persistent elevation in estrogen in obese, postmenopausal women exacerbates age-related vascular damage to a greater extent than in males. Astrocyte expression is also sensitive to fluctuations in estrogen (Fuente-Martin et al., 2013) and greater numbers of GFAP-positive astrocytes have been previously reported in the hippocampus and the posterodorsal portion of the medial amygdala of adult male versus female mice (Conejo et al., 2005; Johnson et al., 2008). Although these reports are consistent with our findings, additional experiments are needed to understand why opposite patterns of perivascular astrocyte expression were observed between male and female offspring.

The relatively weak impact of prenatal HF exposure alone (i.e., HF/C) in female offspring in our study supports previous reports that male offspring are more likely than female offspring to develop behavioral, cognitive and epigenetic brain changes following exposure to a perinatal HF diet (Bilbo and Tsang, 2010; Vucetic et al., 2010; Edlow et al., 2016; Graf et al., 2016; Deardorff et al., 2017; Glendining and Jasoni, 2019). Notably however, female HF/C mice demonstrated larger microglia and higher numbers of phagocytic microglia and macrophages than HF/C male offspring. Sexual dimorphism in microglia number and activation has been described, with microglia in adult female animals reported to have a more inflammatory profile than males (Nissen, 2017), which has been hypothesized to contribute to the increased prevalence of neurodegenerative diseases such as Multiple Sclerosis and AD in women (Nissen, 2017). However, previous studies have reported lower expression of pro-inflammatory and higher levels of anti-inflammatory cytokines in the brains of P21 and young adult female offspring born to HF-fed mothers compared to male (Bilbo and Tsang, 2010; Graf et al., 2016). Therefore, further investigation is needed to determine how perinatal HF exposure impacts on the inflammatory profile across the lifespan of male and female offspring.

We also find it notable that the degree of IgG extravasation, loss of perivascular astrocyte contact and degree of inflammation was significantly greater in female HF/HF mice compared to HF/HF males. Moreover, the difference in astrocyte endfoot contact, parenchymal IgG coverage and Iba1 expression was also higher between C/HF and HF/HF females than males. These data suggest that possible compensations made by male mice during early life exposure to a HF diet are advantageous in the context of a chronic, postnatal obesogenic environment, but that these same adaptations are either not made or are deleterious in the same context in aged females.

Although the current study did not include behavioral analyses, a range of cognitive and pathological effects have been reported in both prenatal and postnatal HF feeding studies. In murine models, HF-diet-induced obesity is associated with impaired cognitive performance and reduced plasticity in both young adult and middle-aged animals (Winocur et al., 2005; Farr et al., 2008; Stranahan et al., 2008). Obesity also accelerates cognitive dysfunction and pathology in AD mouse models (Moser and Pike, 2017; Rollins et al., 2019). In humans, childhood, adolescent and adult obesity is associated with reduced executive function and lower global cognition (Wang et al., 2016), while mid-life obesity is associated with more rapid deterioration in cognitive function and increased risk of developing AD (Kivipelto et al., 2002). However, the association between body-mass index and cognitive function is weaker in old age (Aslan et al., 2015) and the relative risk for obesity-related stroke is lower in older individuals (Emerging Risk Factors Collaboration Wormser et al., 2011). These findings support the influence of early life programing on the long-term health of the brain. To that end, adult offspring exposed to a HF diet during the perinatal period have been reported to have higher levels of anxiety (Bilbo and Tsang, 2010; Peleg-Raibstein et al., 2012; Sasaki et al., 2013; Kang et al., 2014) and depression (Lin et al., 2015; Balsevich et al., 2016) and impaired memory performance (White et al., 2009; Can et al., 2012; Page et al., 2014; Lepinay et al., 2015), although opposite findings have also been reported (Clouard et al., 2016; Johnson et al., 2017; Val-Laillet et al., 2017). Impairments in memory acquisition and retention and increased pathology have also been noted in two mouse models of AD in 12–13 months offspring exposed to a high fat diet during gestation and lactation (Martin et al., 2014; Nizari et al., 2016), suggesting that perinatal HF exposure may also contribute to the pathogenesis of AD. As suggested above, the impact of maternal obesity appears to differentially affect male and female offspring. Male, but not female offspring born to HF-fed dams have been reported to show impairments on the novel object recognition test (Graf et al., 2016), while young adult female, but not male, macaques born to mothers fed a high fat diet showed increased anxiety to novel and threatening objects (Sullivan et al., 2010). Recently, a multi-generational study reported that second and third generation female, but not male offspring born to HF-fed mothers showed impairments in executive function and memory (Sarker and Peleg-Raibstein, 2018). As rates of maternal obesity continue to rise, the current findings may have implications for the future incidence and management of age-related neurovascular diseases, such as stroke and AD, that are more common in obese, post-menopausal women (Lisabeth and Bushnell, 2012; Christensen and Pike, 2015).

## Data Availability Statement

The datasets generated for this study are available on request to the corresponding author.

## Ethics Statement

The animal study was reviewed and approved by Open University Animal Welfare and Ethics Review Board.

## Author Contributions

LC, CJH and CAH designed the experiments which were carried out by LC, SN, and CAH. CJH and CAH wrote and edited the manuscript.

## Conflict of Interest

The authors declare that the research was conducted in the absence of any commercial or financial relationships that could be construed as a potential conflict of interest.
