# Supplementary figures and images for: Pre- and Post-natal High Fat Feeding Differentially Affects the Structure and Integrity of the Neurovascular Unit of 16-Month Old Male and Female Mice
Source: Front Neurosci. 2019 Oct 2;13:1045. doi: 10.3389/fnins.2019.01045 (PMC6783577; doi:10.3389/fnins.2019.01045)

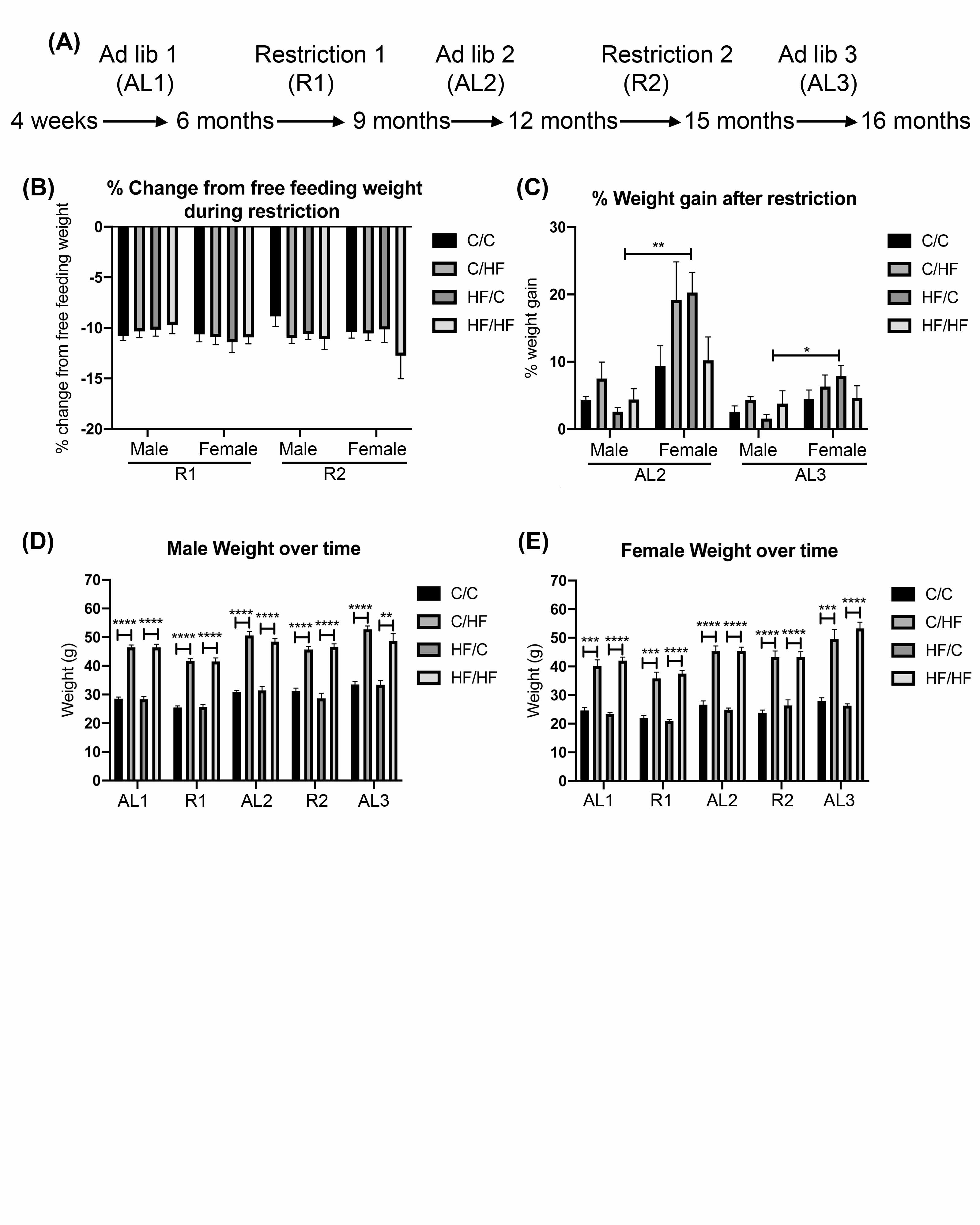

Supplement: Supplementary file 1 [file Image_1.JPEG]

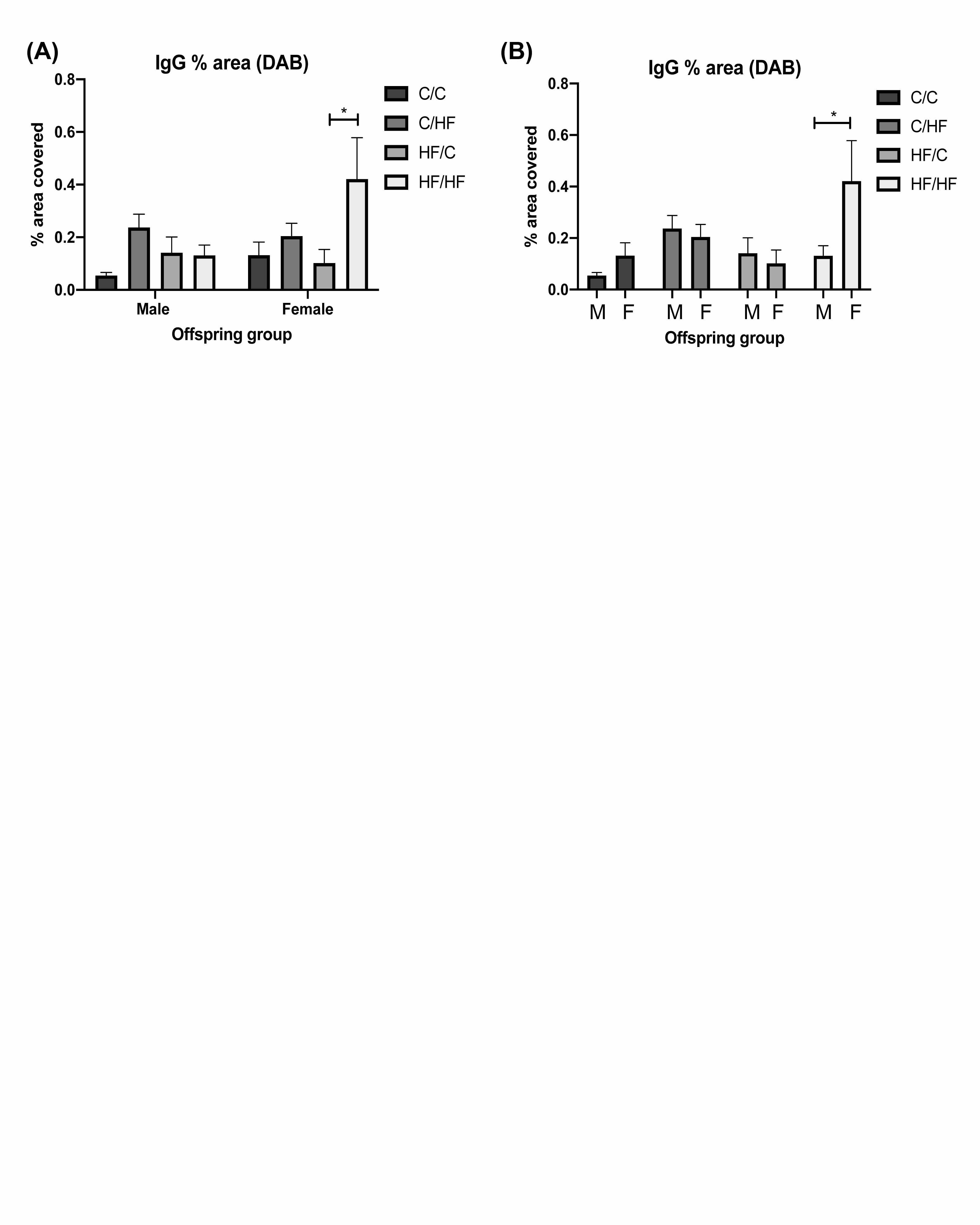

Supplement: Supplementary file 2 [file Image_2.JPEG]

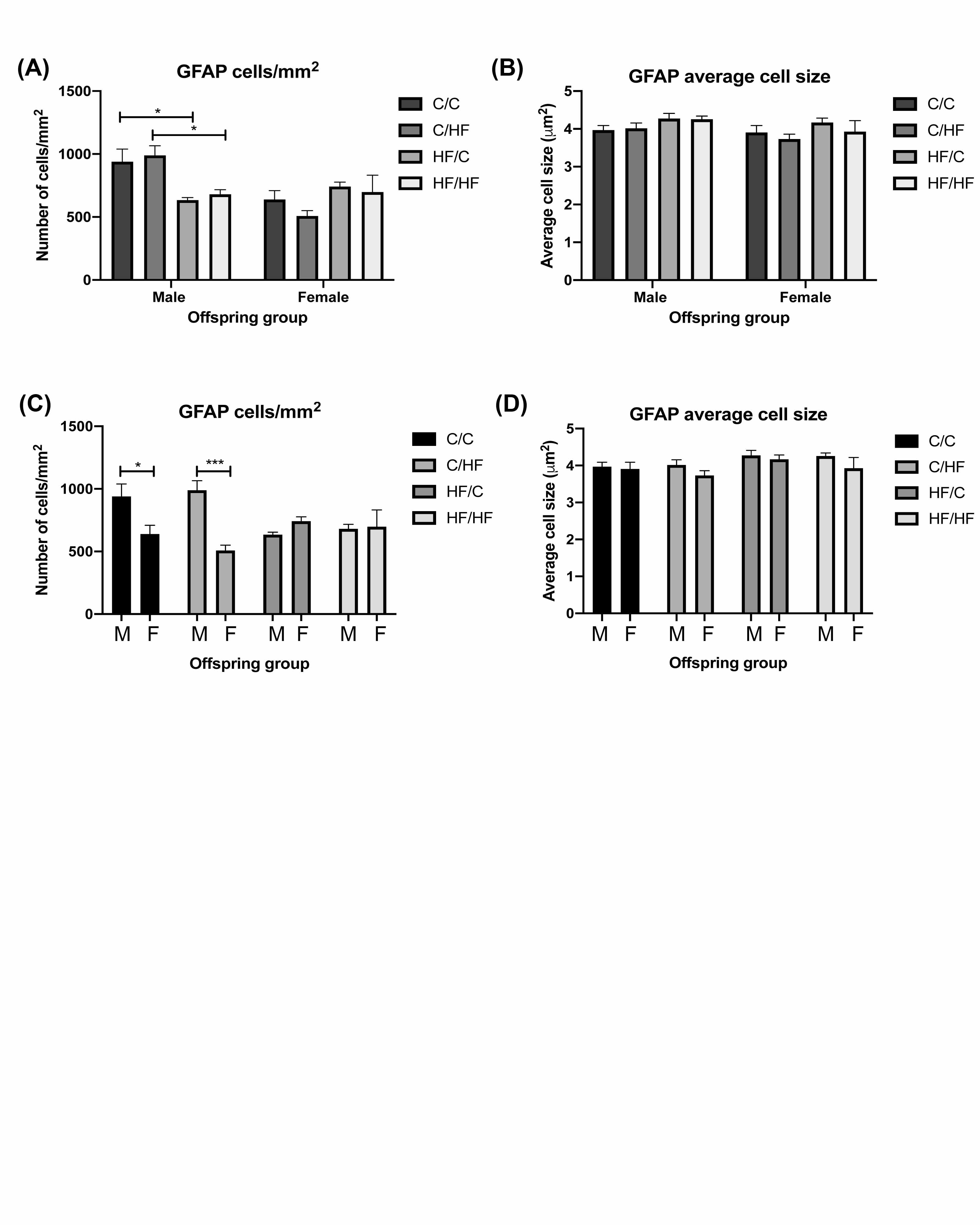

Supplement: Supplementary file 3 [file Image_3.JPEG]
