# Supplementary material for: Pre- and Post-natal High Fat Feeding Differentially Affects the Structure and Integrity of the Neurovascular Unit of 16-Month Old Male and Female Mice
Source: Front Neurosci. 2019 Oct 2;13:1045. doi: 10.3389/fnins.2019.01045 (PMC6783577; doi:10.3389/fnins.2019.01045)
Supplement: Supplementary file 4 [file Table_1.DOCX]

**Supplemental Figure Legend**

**Figure 1 Schedule of food restriction and ab libitum consumption and weight of offspring over the 16-month experiment.** (A) Timeline of the periods of ad libitum (AL) and restricted (R) food consumption of male and female C/C, C/HF, HF/C and HF/HF offspring over the course of the 16-month experiment. All animals underwent the same schedule of food consumption and restriction. (B) Percent change in offspring body weight during periods of food restriction compared to weight during ad libitum feeding. (C) Percent weight gain of male and female offspring during ad libitum feeding after periods of food restriction. (D and E) Body weight of male (D) and female (E) C/C, C/HF, HF/C and HF/HF offspring over the periods of ad libitum and food restriction. Data represent mean ±SEM. **p*<0.05, ***p*<0.01, ****p*<0.001, two-way ANOVA with Holm-Sidak post-hoc test.

**Figure 2 Evaluation of IgG extravasation in the hippocampus of aged offspring using DAB immunohistochemistry.** (A and B) Quantification of hippocampal area positive for IgG extravasation as evaluated by single labeling immunohistochemistry in 16-month old male and female C/C, C/HF, HF/C and HF/HF mice by diet group (A) and by sex (B). Data represent mean ±SEM. **p*<0.05, two-way ANOVA with Holm-Sidak post-hoc test.

**Figure 3 Evaluation of GFAP expression in the hippocampus of aged male and female offspring.** (A-D) Quantification of cell count (A, C) and average size (B, D) of GFAP-positive astrocytes in the hippocampus of 16-month old male and female C/C, C/HF, HF/C and HF/HF mice. Data represent mean ±SEM. **p*<0.05, ****p*<0.001, two-way ANOVA with Holm-Sidak post-hoc test.
