# Supplementary material for: Pre- and Post-natal High Fat Feeding Differentially Affects the Structure and Integrity of the Neurovascular Unit of 16-Month Old Male and Female Mice
Source: Front Neurosci. 2019 Oct 2;13:1045. doi: 10.3389/fnins.2019.01045 (PMC6783577; doi:10.3389/fnins.2019.01045)
Supplement: Supplementary file 5 [file Table_2.DOCX]

**Supplemental Table 1** Diet Composition

|  | Control diet | High fat diet |
| --- | --- | --- |
| **Ingredients (%)** |  |  |
| Casein | 21.5 | 26.51 |
| Choline bitartrate | 0.24 | 0.30 |
| L-Cystine | 0.32 | 0.40 |
| Lard | 0 | 18 |
| Rice Starch | 32.44 | 18.43 |
| Cellulose | 5 | 6.16 |
| Soya oil | 3.5 | 4.32 |
| Sucrose | 32.5 | 20.3 |
| Mineral mix | 3.5 | 4.3 |
| Vitamin mix | 1 | 1.2 |
| **Total** | 100% | 100% |
|  |  |  |
| **Fatty acids (%)** |  |  |
| C12:0 (lauric acid) | 0.07 | 0.03 |
| C14:0 (myristic acid) | 0.12 | 0.29 |
| C16:0 (palmitic acid) | 0.22 | 4.04 |
| C18:0 (steric acid) | 0.13 | 1.8 |
| C14:1 (myristoleic acid) | 0.01 | 0.02 |
| C16:1 (palmitoleic acid) | 0.07 | 0.03 |
| C18:1 (oleic acid) | 0.66 | 6.06 |
| C18:2 (linoleic acid) | 1.26 | 3.53 |
| C18:3 (linolenic acid) | 0.22 | 0.37 |
| C20:4 (arichidonic acid) | 0.01 | 0.01 |
| C22:5 (clupanodonic acid) | 0 | 0 |
|  |  |  |
| **Amino acids (%)** |  |  |
| Arginine | 0.55 | 0.69 |
| Lysine | 1.11 | 1.37 |
| Methionine | 0.43 | 0.53 |
| Cysteine | 0.35 | 0.42 |
| Tryptophan | 0.16 | 0.19 |
| Histidine | 0.4 | 0.49 |
| Threonine | 0.61 | 0.75 |
| Isoleucine | 0.88 | 1.09 |
| Leucine | 1.33 | 1.64 |
| Phenylalanine | 0.73 | 0.9 |
| Valine | 1.06 | 1.3 |
| Tyrosine | 0.73 | 0.9 |
| Taurine | 0 | 0 |
| Glycine | 0.7 | 0.85 |
| Aspartic acid | 0.99 | 1.22 |
| Glutamic acid | 2.87 | 3.53 |
| Proline | 1.2 | 1.47 |
| Serine | 0.66 | 0.82 |
| Alanine | 0.56 | 0.69 |
|  |  |  |
| **Macro minerals (%)** |  |  |
| Calcium | 0.46 | 0.59 |
| Total Phosphorus | 0.18 | 0.35 |
| Sodium | 0.12 | 0.15 |
| Chloride | 0.22 | 0.26 |
| Potassium | 0.46 | 0.42 |
| Magnesium | 0.06 | 0.08 |
|  |  |  |
| **Micro minerals** |  |  |
| Iron (mg/kg) | 44.99 | 55.7 |
| Copper (mg/kg) | 6.67 | 8.22 |
| Manganese (mg/kg) | 10.13 | 12.22 |
| Zinc (mg/kg) | 54.63 | 64.67 |
| Cobalt (μg/kg) | 0 | 0 |
| Iodine (μg/kg) | 194.04 | 238.95 |
| Selenium (μg/kg) | 138.13 | 185.09 |
|  |  |  |
| **Vitamins** |  |  |
| Vitamin A (iu/kg) | 3758.61 | 4628.65 |
| Vitamin D (3) (iu/kg) | 2151.81 | 2822.23 |
| Vitamin E (iu/kg) | 74.09 | 94.46 |
| Vitamin B1 (mg/kg) | 5.61 | 5.73 |
| Vitamin B2 (mg/kg) | 4.86 | 5.98 |
| Vitamin B6 (mg/kg) | 6.53 | 6.7 |
| Vitamin B12 (μg/kg) | 23.49 | 28.93 |
| Vitamin C (mg/kg) | 0 | 0 |
| Vitamin K (mg/kg) | 0.68 | 0.89 |
| Folic acid (mg/kg) | 1.88 | 2.22 |
| Nicotinic acid (mg/kg) | 27.91 | 34.37 |
| Pantothetic acid (mg/kg) | 13.74 | 17.33 |
| Choline (mg/kg) | 926.94 | 1248.4 |
| Inositol (mg/kg) | 0 | 0 |
| Biotin (μg/kg) | 187.93 | 231.43 |
